# Identification and validation of reference genes for real-time RT-PCR in *Aphelenchoides besseyi*

**DOI:** 10.1007/s11033-020-05547-8

**Published:** 2020-05-28

**Authors:** Junyi Li, Zixu Zhang, Chunling Xu, Dongwei Wang, Mei Lv, Hui Xie

**Affiliations:** Laboratory of Plant Nematology and Research Center of Nematodes of Plant Quarantine, Department of Plant Pathology/Guangdong Province Key Laboratory of Microbial Signals and Disease Control, College of Agriculture, South China Agricultural University, 483 Wushan Road, Guangzhou, 510642 Guangdong People’s Republic of China

**Keywords:** *Aphelenchoides besseyi*, RT-qPCR, Reference gene, Expression, *18S rRNA*

## Abstract

Fragments of four candidate reference genes of *Aphelenchoides besseyi*, including *actin*, glyceraldehyde-3-phosphate dehydrogenase (*GAPDH*)*,* ubiquitin conjugating-3 enzyme (*UBC*) and alpha-tubulin (*α-tubulin*) were cloned from the transcriptome database of *A*. *besseyi*. The expression level of these four candidate reference genes and a commonly used reference gene of *A. besseyi* (*18S rRNA*) in three experimental conditions, including the four life stages (female, male, juvenile and egg) of two populations and the mixed-stage nematodes of four populations with different origins and hosts were analyzed by RT-qPCR. The expression stability of the five candidate reference genes under the three experimental conditions was analyzed by ΔCt, geNorm, NormFinder and RefFinder respectively. The analysis results of ΔCt, geNorm, NormFinder and RefFinder all indicated that *UBC* was the gene with the highest average ranking of stability. In conclusion, the expression stability of *UBC* was optimal under the three experimental conditions, indicating that *UBC* could be used as a suitable reference gene instead of *18S rRNA* in the RT-qPCR analysis for *A. besseyi.*

**Electronic supplementary material:**

The online version of this article (10.1007/s11033-020-05547-8) contains supplementary material, which is available to authorized users.

## Introduction

Quantitative real time PCR (qPCR) is a robust nucleic acid quantitative technique developed on the basis of traditional PCR technology, in which the fluorescent group is added into the PCR reaction system to detect the change of the product quantity by detecting fluorescence intensity change [1]. Compared with traditional PCR, it’s quantitative with an enormous dynamic window. It has become one of the most common techniques in gene expression quantification and transcriptome analysis. A reference gene, also known as a housekeeping gene, is a gene that is expressed in all cells and its expression level is little affected by environmental factors and remains consistent among growth stages, organs and tissues (Pfaffl [2]). When using reverse-transcription qPCR (real-time RT-PCR, RT-qPCR) to analyze the relative quantitative expression of target genes, the incorporation of a reference gene is helpful to correct for any deviations in the initial template amount, RNA quality and enzymatic reaction efficiency among different tissues and cells. By using the reference genes to normalize and standardize the expression level of the target gene, errors in the experimental process can be reduced, thus improving the reliability of the results [3, 4]. The ideal reference gene has constant expression under various experimental conditions, such as different life stages or different populations of nematodes. However, recent studies have shown that there is no perfect reference gene, as experimental conditions change, some traditional reference gene’s expression level change as well. Therefore, under a specific set of experimental conditions, the use of an inappropriate reference gene can lead to inaccurate quantitative results [5, 6]. In recent years, some studies have been conducted to identify appropriate reference genes for different kinds of nematodes. Twelve reference genes were selected to determine their expression stability in *Caenorhabditis elegans* under different experimental conditions. The results showed that *cdc-42, pmp-3* and *Y45F10D.4* were relatively stable reference genes (Hoogewijs et al. [7]). The expression stability of 11 reference genes was studied between females and males of different genotypes of *Haemonchus contortus* and *gpd*, *ama* and *far* were identified as good reference genes [8]. Eleven candidate reference genes were chosen from RNA-seq data of *Globodera rostochiensis* to identify a reliable set of reference genes to study gene expression, three genes including *GR*, *PMP-3* and *aaRS* were found to be stable in different stages of *G.rostochiensis* (Sabeh et al. [9]). *eIF5a* were identified as a suitable reference gene that could be expressed stably in different developmental stages and different populations of *Radopholus similis* [10]*.* Such studies confirmed that not all traditional reference genes are suitable for a qPCR relative quantitative analysis under all experimental conditions; the most suitable reference gene may change as the experimental conditions change. At present, there are no other reports on the reference gene screening of plant nematodes except *R. similis* and *G. rostochiensis*, so the research on this field remain further study.

*Aphelenchoides besseyi*, which is widely distributed in most rice-growing areas throughout the world, is a foliar nematode that feeds endoparasitically or ectoparasitically on the above-ground parts of plants and is one of the most damaging plant nematodes to rice [11]. *A. besseyi* also endangers 35 genera of higher plants, including strawberries, maize and chestnut, and it is considered to be one of the ten most important plant parasitic nematodes [12]. At present, there are few studies on the reference genes of *A. besseyi,* and only *18S rRNA* was used as reference gene in RT-qPCR analysis [13, 14]. However, there are several arguments against the use of *18S rRNA* as a suitable reference gene. First, the expression level of *18S rRNA* in cells is too high to deduct the baseline value for a target gene with low expression level in RT-qPCR experiments, which might lead to inaccurate results. Second, oligo dt primers are commonly used in reverse transcription of target genes, while random primers must be used in reverse transcription experiments of *18S rRNA* due to the absence of polyA, which may cause deviation due to different reverse transcription efficiency. Third, the gene type of the reference genes and target genes should be close; however, *18S rRNA* is a kind of ribosomal RNA gene whereas most target genes are mRNA genes. The effect of the partial degradation of RNA on the expression level of *18S rRNA* is typically less than that of the target gene, which makes *18S rRNA* unsuitable for monitoring the target gene [6, 15–17]. Fourth, some nematode species of Aphalenchoides, in particular *A. besseyi* and the closely related *A. fujianensis* has been reported as having variation in ribosomal subunit copy number between species and populations [18, 19], suggesting that the expression level of *18S rRNA* may vary greatly between different populations. Therefore, it’s necessary to find a better reference gene to replace *18S rRNA* for *A. besseyi* expression studies.

ΔCt [20], geNorm [21], NormFinder [22] and RefFinder [23] are the four main types of software used to analyze the stability of reference genes. ΔCT method calculates the expression quantity (Q) according to the formula Q = 2^ΔCt^, which will be a basis for the software to rank the expression stability of each reference gene [20]. For geNorm, the default value of the software M = 1.5 is used to calculate average expression stability value (M) of each reference gene. If the M value of the candidate reference gene is less than 1.5, it can be considered as an available reference gene. In addition, geNorm determines the effect of introducing new reference genes by pairwise variation analysis (V_n_/V_n + 1_ and determines the optimal number of reference genes; geNorm software uses V_n_/V_n + 1_ = 0.15 as the cut-off value, if V_n_/V_n + 1_ < 0.15, it means that (*n*) genes have been stable as a combination with no need for introducing the (n + 1) reference genes. However, the geNorm operation manual states that the default cut-off value is not fixed and can be adjusted slightly according to the biological and experimental conditions [21]. Because the value V_n_/V_n + 1_ were all greater than 0.15 under one of the three experimental conditions in this study, the cut-off value V_n_/V_n + 1_ was set to 0.2 and the optimal number of reference genes is selected under the condition of V_n_/V_n + 1_ < 0.2. Similar to geNorm, NormFinder can calculate the average expression stability value M of each candidate reference gene, the smaller the M value, the higher the expression stability of a reference gene [22]. The online program RefFinder (https://www.heartcure.com.au/reffinder/?type=reference) integrates the evaluation results of multiple reference gene analysis softwares such as ΔCt, geNorm, and NormFinder to give each analysis method a certain weight. A higher ranking indicates a higher stability of a reference gene [23].

In recent years, using transcriptome database to screen reference genes has been proved to be an effective method, which has been applied to yeast [24], fungi [25], animals [26, 9, 27] and plants [28]. In our previous work [29], in order to understand the developmental and reproductive characteristics of *A. besseyi* at the molecular level and the mechanism of interaction between *A. besseyi* and host plants, we have generated a transcriptome database (accession number: SRR4002929 and SRR4002930) consisting of two populations of *A. besseyi* with different pathogenicity. In this study, the four commonly used reference genes *actin, GAPDH, UBC* and *α-tubulin* were cloned from this transcriptome database. The expression stability of these four candidate genes and *18S rRNA* in four life stages of two populations and mixed-stage of four populations of *A. besseyi* were studied by four commonly used reference gene screening software including ΔCt, geNorm, NormFinder and RefFinder. The results of this study confirmed that *UBC* is an ideal reference gene in RT-qPCR experiments of *A. besseyi*, which laid a solid foundation for accurate quantification of *A. besseyi* genes.

## MATERIALS AND METHODS

### Nematode

Four populations of *A. besseyi* (Table S1) used in this study were collected and identified by Plant Nematology Laboratory, South China Agricultural University. *A. besseyi* was preserved and cultured on excised carrot (*Daucus carota*) disks in Petri dishes at 25 ℃ in the dark (0-h light/24-h dark photoperiod) as described previously [30].

### RNA isolation and reverse transcription

The total RNA of 12 samples, which include four mixed-stage nematodes samples from four populations (N10, S24, X8 and YQ) (each sample contained 1250 mixed-stage nematodes), six samples of single developmental stage (female, male and juvenile) from populations N10 and S24 (each sample contained 1000 nematodes), and two egg samples of populations N10 and S24 (each sample contained 3000 eggs), were extracted according to the instructions of the miRNeasy micro kit (Qiagen, USA), respectively. Each sample was replicated three times. The concentration of total RNA of each sample was determined using a NanoDrop2000c spectrophotometer (Thermo Fisher Scientific, USA) and its integrity and quality were confirmed by 1% agarose gel electrophoresis. 200 ng total RNA of each sample was reverse-transcribed into cDNA by TransScript One-Step gDNA Removal and cDNA Synthesis Super Mix Kit (Trans, China) according to the manufacturer’s protocol. All templates were stored at − 20 °C until use.

### Cloning and bioinformatic analysis of the candidate reference genes

Referring to the transcriptome data (accession number: SRR4002929 and SRR4002930) generated by our laboratory, the fragments of four candidate reference genes *actin, GAPDH, UBC* and *α-tubulin* were cloned*.* The sequence of *18S rRNA* (AY508035) was downloaded from NCBI. Specific primers were designed for cloning the fragments of the four candidate reference genes of *A. besseyi* by Primer Premier 5.0 (Primer Biosoft, USA) (Table S2)*.* PCR was performed with a cDNA template of the mixed-stage nematodes of N10. The reaction procedure in a 20 μL volume that included 2.0 μL template cDNA, 200 nM of gene-specific primers and 10 μL of 2 × Taq master mix (GenStar, China) was as follows: 94 °C for 2 min; 40 cycles of 94 °C for 30 s, 58 °C for 30 s and 72 °C for 2 min; and a final elongation step at 72 °C for 5 min. The purified products were sequenced and subjected to BLAST search via the NCBI website, and ORFfinder (https://www.ncbi.nlm.nih.gov/orffinder/) was used to predict their corresponding amino acid sequences.

### Primer design and RT-qPCR

The RT-qPCR primers were designed based on conserved sequence regions of the five candidate reference genes by Primer Premier 5.0 (Primer Biosoft, USA) and Primer-blast (www.ncbi.nlm.nih.gov/tools/primer-blast/) (Table S3). The RT-qPCR analysis was performed with each reaction mixture in a 20 μL volume containing 250 nM of each primer, 2 µL of diluted cDNA templates and 10 µL of AceQ qPCR SYBR® greenMaster Mix (Vazyme, China) on Bio-Rad CFX-96 Real-Time PCR system (Bio-Rad, USA). The thermal cycling conditions were as follows: 95 °C for 60 s, followed by 40 cycles of 95 °C for 15 s and 60 °C for 60 s. Melting curve analysis was performed after 40 cycles to verify the reaction specificity. Then, the RT-qPCR products were visualized by 2% agarose gel. A standard curve was created by a fivefold dilution series of the mixed cDNA from all samples, and PCR amplification efficiency (E) values of all primer pairs were calculated based on the standard curves according to the formula E = [10 (− 1/slope) − 1] × 100% [6]. Each RT-qPCR analysis was performed with three biological replicates. In addition, a non-template control was included for each replicate with RNase—free water instead of cDNA templates.

### Data analysis

To homogenize the runs, the qPCR threshold used to determine threshold values (Ct) was set the same fluorescence value (RFU). The Ct value for each amplification curve was determined by the Bio-Rad CFX-96 Manager software (Bio-Rad, USA). The stability of the five candidate reference genes under the three experimental conditions, including the four life stages (female, male, juvenile and egg) of populations N10, the four life stages (femal, male, juvenile and egg) of populations S24 and the mixed-stage nematodes of all four populations were evaluated by ΔCt [20], geNorm [21], NormFinder [22] and RefFinder [23]. All raw Ct values were subjected to analysis by one-way ANOVA and tested for differences between groups at a 5% level using Turkey Test.

## Results

### Cloning and bioinformatic analysis of the candidate reference genes

Using the mixed cDNA of all samples as a template, the specific fragments of *actin* (876 bp), *GAPDH* (563 bp), *UBC* (615 bp) and *α-tubulin* (1104 bp) were amplified by PCR using specific primers of the four candidate reference genes (Fig. S1). The amplified products were sequenced and the results showed that the size of all sequences was consistent with expectations. The amino acid sequences encoded by the fragment of the each candidate reference gene were predicted by ORFfinder (https://www.ncbi.nlm.nih.gov/orffinder/), respectively. The nucleic acid sequences and predicted amino acid sequences of each reference gene fragment were compared with that of other nematode genes in NCBI by performing BLAST analysis. The results showed that the similarity of the nucleic acid sequences and predicted amino acid sequences between the four candidate reference genes of *A. besseyi* and corresponding genes of other nematodes were all greater than 70% (Table S4). Among them, the amino acid sequence similarity between *actin* of *A. besseyi* and that of *Bursaphelenchus xylophilus* and *Caenorhabdit elegans* has reached 100%. Further analysis showed that the predicted amino acid sequences of the four candidate reference genes all have conserved domains encoded by the corresponding genes (Fig. S2). Therefore, the *actin, GAPDH, UBC* and *α-tubulin* gene fragments amplified in this study were *actin* (KY992856)*, GAPDH* (KY992853)*, UBC* (KY992854) and *α-tubulin* (KY992855) gene fragments of *A. besseyi*.

### Primers analysis of the candidate reference genes in RT-qPCR

The melting curves of *actin*, *GAPDH*, *UBC*, *α-tubulin* and *18S rRNA* gene generated by RT-qPCR were all single peaks (Fig. S3), indicating that the RT-qPCR primers of the five candidate reference genes had good specificity and no dimer amplification. The size of the RT-qPCR products of the five candidate reference genes confirmed by 1% agarose gel electrophoresis all matched expectations (Fig. S4), which further validated the high specificity of the RT-qPCR primers.

### Expression of candidate reference genes

**Fig. 1:**
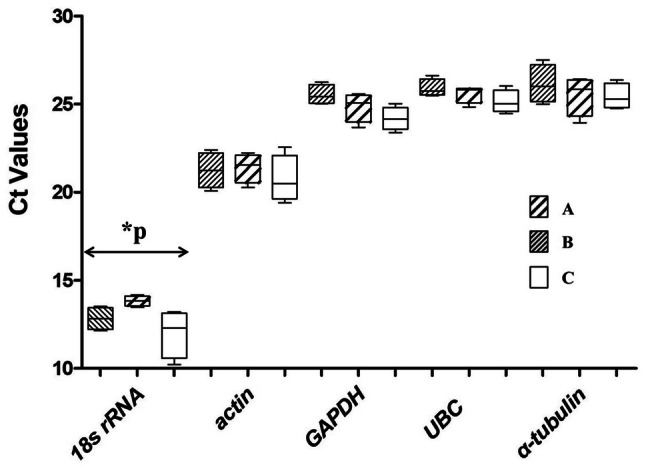
Range of all Ct values of the five candidate reference genes of *Aphelenchoides besseyi*. **a** Four life stages of N10 population. **b** Four life stages of S24 population. C: Four different populations. 75% of the Ct values are in the "box" range. 25% of the Ct values are in the "vertical line" range. The “short lines” represent the maximum and minimum Ct values. The "horizontal line within the square" represents the median Ct value. *P indicates significant differences between groups (P < 0.05)

Ct value is closely related to gene expression, and the higher Ct value, the lower the gene expression. In order to compare the expression level of the five candidate reference genes, expression of these genes in 12 samples divided into three groups (the three experimental conditions), including a group consisting of mixed-stage nematodes of four populations N10, S24, X8 and YQ, a group consisting of four life stages (female, male, juvenile and egg) of population N10 and a group consisting of four life stages (female, male, juvenile and egg) of population S24 were detected by RT-qPCR. The results showed the expression level of the five candidate reference genes were not consistent across all samples (Fig. 1). The Ct values of *18S rRNA* ranged from 10.22 to 14.18 in 12 samples and significantly differed among the groups (P = 0.0483). The Ct values of *actin, GAPDH, UBC* and *α-tubulin* ranged from 19.40 to 22.55, 23.8 to 26.24, 24.46 to 26.60 and 23.94 to 27.50 in all 12 samples, respectively. But there were no significant difference among the groups for the four genes (P = 0.669, P = 0.645, P = 0.207, P = 0.572). Among the five genes, *18S rRNA* and *α-tubulin* are the genes with the highest and lowest expression level respectively, while *actin*, *GAPDH* and *UBC* are the genes with moderate expression level. The average Ct values of the five genes were 25.68(*α-tubulin*), 12.89(*18S rRNA*), 21.12(*actin*), 24.85(*GAPDH*) and 25.53(*UBC*). An ideal reference gene should have a moderate expression level with a Ct value between 15 and 30 [31, 32], the high or low expression of a reference gene will affect the accuracy of quantitative results [21]. Among the five genes, *UBC* showed the lowest variation in expression among the 12 samples with a difference of 2.14 between the maximum and minimum CT values. In contrast, *18S rRNA* showed the highest variation in expression among the 12 samples with a difference of 3.96 between the maximum and minimum CT values, and the difference was significant among groups (P < 0.05). The results showed that the commonly used reference gene *18S rRNA* of *A.besseyi* is not the best choice in the RT-qPCR analysis of the three experimental conditions in this study. Further analysis is still needed to screen out the optimal reference gene.

### Stability of reference genes

#### Δ Ct method analysis

The expression stability of the five candidate reference genes of *A. besseyi* were analyzed using the ΔCt method. The results showed that under the three experimental conditions, the expression stability of the five candidate reference genes was ordered as follows: *UBC* > *actin* > *18S rRNA* > *α-tubulin* > *GAPDH*; *18S rRNA* > *UBC* > *GAPDH* > *actin* > *α-tubulin*; and *UBC* > *GAPDH* > *α-tubulin* > *actin* > *18S rRNA* (Table 1). Therefore, the average stability ranking of the five reference genes in descending order was *UBC* (1.33) > *18S rRNA* (3) > *actin* (3.33) = *GAPDH* (3.33) > *α-tubulin* (4) (Table 1). The stability of *18S rRNA* ranked first and third respectively in population S24 and population N10, while it ranked last between populations. When the data of mixed-population were excluded from analysis, the average stability ranking was *UBC* (1.5) > *18S rRNA* (2) > *actin* (3) > *GAPDH* (4) > *α-tubulin* (4.5). Although the stability ranking of *18S rRNA* was still lower than that of *UBC*, its average ranking in this case has risen by 1 place, indicating the expression level of *18S rRNA* varies more between populations than within a certain population. The results showed that *UBC* was the most stable reference gene; *18S rRNA*, *actin* and *GAPDH* were moderately stable reference genes; *α-tubulin* was the least stable reference gene.

**Table 1:** Stability ranking of the five candidate reference genes of *Aphelenchoides besseyi* calculated by ΔCt

Stability ranking	Four stages in population N10		Four stages in population S24		Mixed-stage nematodes of the four populations		Average ranking	
	Genes	Stability value	Genes	Stability value	Genes	Stability value	Genes	Ranking
1	*UBC*	0.676	*18S rRNA*	0.793	*UBC*	0.544	*UBC*	1.33
2	*actin*	0.685	*UBC*	0.800	*GAPDH*	0.580	*18S rRNA*	3
3	*18S rRNA*	0.713	*GAPDH*	0.955	*α-tubulin*	0.645	*actin*	3.33
4	*α-tubulin*	0.722	*actin*	1.016	*actin*	0.808	*GAPDH*	3.33
5	*GAPDH*	1.059	*α-tubulin*	1.343	*18S rRNA*	1.031	*α-tubulin*	4

#### geNorm analysis

**Fig. 2:**
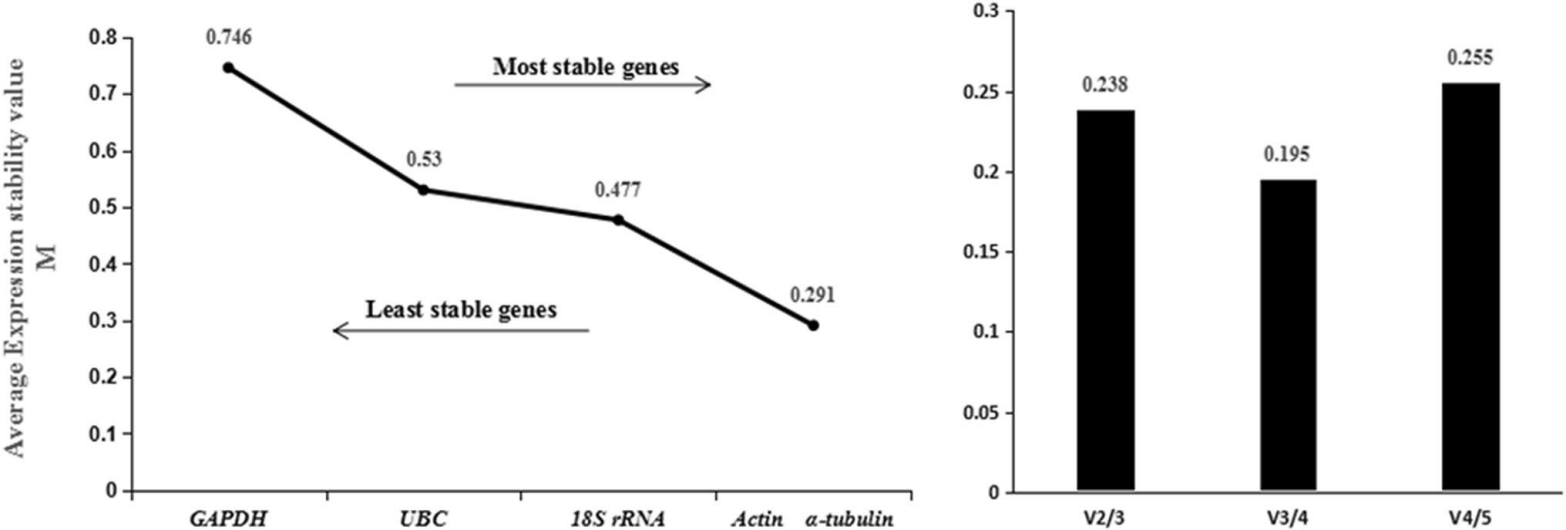
geNorm analysis of the five candidate reference genes of four life stages in population N10

**Fig. 3:**
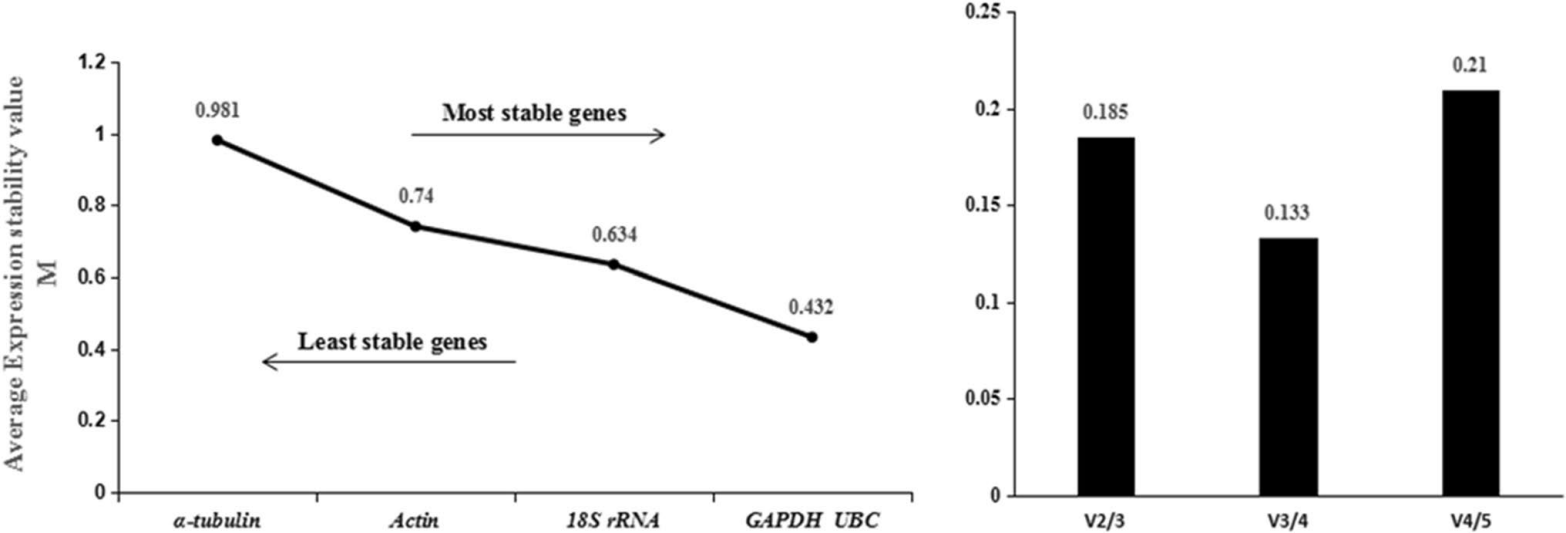
geNorm analysis of the five candidate reference genes of four life stages in population S24

**Fig. 4:**
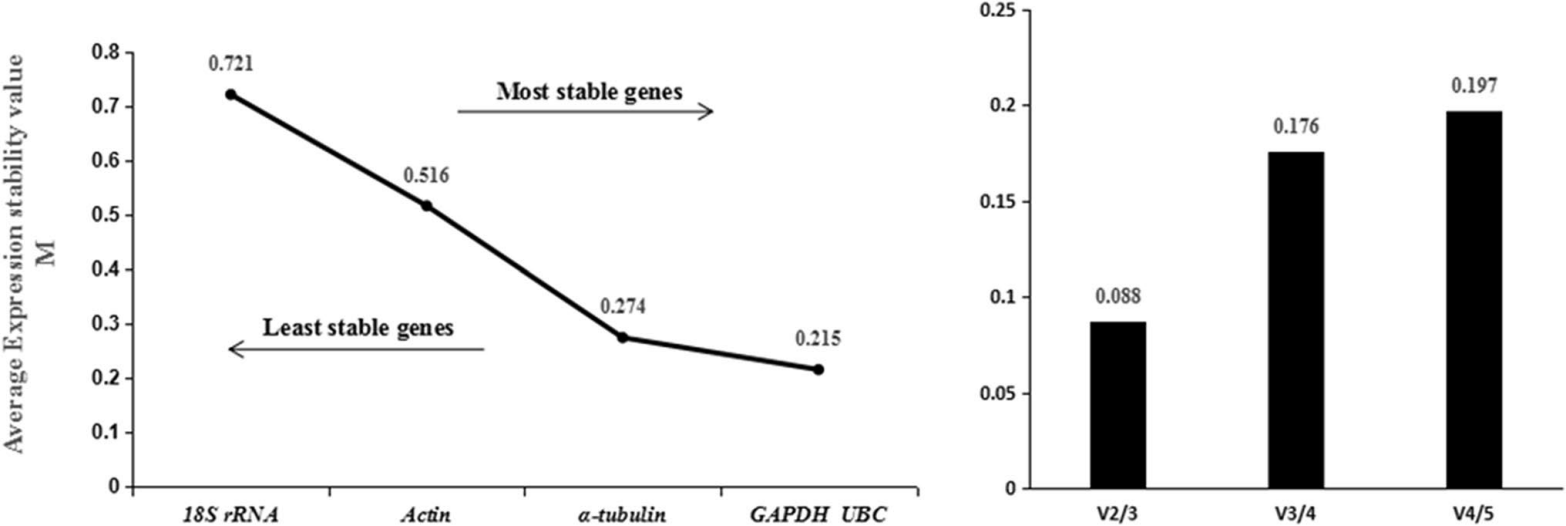
geNorm analysis of the five candidate reference genes of mixed-stage nematodes of four populations

The results of geNorm analysis showed that the M values of the five candidate genes were all less than 1.5 in all three experimental conditions, indicating the five genes were all evaluated as available reference genes. For the four life stages of population N10, the M values of the five candidate genes followed the order *GAPDH* > *UBC* > *18S rRNA* > *α-tubulin* = *actin* (Fig. 2), indicating the stability ranking of the five genes followed the order *actin* = *α-tubulin* > *18S rRNA* > *UBC* > *GAPDH* in this situation. For the four life stages of population S24, the M values of the five candidate genes followed the order *α-tubulin* > *actin* > *18S rRNA* > *GAPDH* = *UBC* (Fig. 3), indicating the stability ranking of the five genes followed the order *UBC* = *GAPDH* > *18S rRNA* > *actin* > *α-tubulin* in this situation*.* For the mixed-stage nematodes of the four populations, the M values of the five candidate genes followed the order *18S rRNA* > *actin* > *α-tubulin* > *GAPDH* = *UBC* (Fig. 4), indicating the stability ranking of the five genes followed the order *UBC* = *GAPDH* > *α-tubulin* > *actin* > *18S rRNA* in this situation*.* Therefore, the average stability ranking of the five reference genes in descending order was *UBC* (2) > *GAPDH* (2.33) > *actin* (3) = *α-tubulin* (3) > *18S rRNA*(3.67). Similar to Δ Ct method results, the stability of *18S rRNA* between populations was lower than that within populations, *18S rRNA* was the third stable gene in both population N10 and S24 as well as the least stable gene between populations. When the data of mixed-population were excluded from analysis, the average stability ranking was *UBC* (2.5) = *actin* (2.5) > *18S rRNA* (3) = *GAPDH* (3) = *α-tubulin* (3). In this case, the average ranking and overall ranking of *18S rRNA* among all genes rose from 3.67 to 3 and 5 to 3 respectively. Furthermore, for the four life stages of population N10, V_3/4_ was 0.195 (Fig. 2), indicating the optimal numbers of reference genes was three (*18S rRNA, actin* and *α-tubulin*) in this situation; for the four life stages of population S24, V_2/3_ was 0.185 (Fig. 3), indicating the optimal numbers of reference genes was two (*GAPDH* and *UBC*) in this situation; for the mixed-stage nematodes of four populations, V_2/3_ was 0.088 (Fig. 4), indicating the optimal numbers of reference genes was two (*UBC* and *GAPDH*) in this situation.

#### NormFinder analysis

The results of NormFinder analysis showed that under the three experimental conditions, the expression stability of the five candidate genes followed the order *UBC* > *18S rRNA* > *actin* > *α-tubulin* > *GAPDH*, *UBC* > *18S rRNA* > *GAPDH* > *actin* > *α-tubulin*, *UBC* > *GAPDH* > *α-tubulin* > *actin* > *18S rRNA*, respectively (Table 2). Therefore, the average stability ranking of the five reference genes in descending order was *UBC* (1) > *18S rRNA* (3) > *GAPDH* (3.33) > *actin* (3.67) > *a-tubulin* (4) (Table 2). Similarly, the stability of *18S rRNA* between populations was lower than that within populations, *18S rRNA* ranked second in both population N10 and S24 but ranked last between populations in terms of stability. When the data of mixed-population were excluded from analysis, the average stability ranking was *UBC* (1) > *18S rRNA* (2) > *actin* (3.5) > *GAPDH* (4) > *α-tubulin* (4.5). Although *18S rRNA*’s overall ranking among genes remained the same, its average ranking rose from 3 to 2 in this case. These results showed that *UBC* was the most stable candidate reference gene; *18S rRNA, GAPDH* and *actin* were moderately stable reference genes, and *a-tubulin* was the least stable candidate reference gene under the three experimental conditions.

**Table 2:** Stability ranking of the five candidate reference genes of *Aphelenchoides besseyi* calculated by NormFinder

Stability ranking	Four stages in population N10		Four stages in population S24		Mixed-stage nematodes of the four populations		Average ranking	
	Genes	Stability value	Genes	Stability value	Genes	Stability value	Genes	Ranking
1	*UBC*	0.156	*UBC*	0.235	*UBC*	0.181	*UBC*	1
2	*18S rRNA*	0.366	*18S rRNA*	0.285	*GAPDH*	0.200	*18S rRNA*	3
3	*actin*	0.512	*GAPDH*	0.719	*α-tubulin*	0.475	*GAPDH*	3.33
4	*α-tubulin*	0.575	*actin*	0.787	*actin*	0.599	*actin*	3.67
5	*GAPDH*	1.012	*α-tubulin*	1.244	*18S rRNA*	0.954	*a-tubulin*	4

#### RefFinder analysis

The stable values of the five reference genes obtained by RefFinder analysis showed that under the three experimental conditions, the expression stability of the five reference genes followed the order *UBC* > *actin* > *18S rRNA* > *α-tubulin* > *GAPDH*, *UBC* > *18S rRNA* > *GAPDH* > *actin* > *α-tubuli*n, *UBC* > *GAPDH* > *α-tubulin* > *actin* > *18S rRNA*, respectively (Table 3). Therefore, the average stability ranking of the five reference genes in descending order was *UBC* (1) > *18S rRNA* (3.33) = *actin* (3.33) = *GAPDH* (3.33) > *a-tubulin* (4) (Table 3). Not surprisingly, although *18S rRNA* ranked second and third in population S24 and N10 respectively, it was rated as the least stable gene between populations again. When the data of mixed-population were excluded from analysis, the average stability ranking was *UBC* (1) > *18S rRNA* (2.5) > *actin* (3) > *GAPDH* (4) > *α-tubulin* (4.5), although it did not affect the overall ranking of each gene, the average ranking of *18S rRNA* rose from 3.33 to 2.5 in this case. According to the ranking, we can confirm that *UBC* is the most stable candidate reference genes; *18S rRNA, actin* and *GAPDH* are moderately stable reference genes, and *a- tubulin* is the least stable reference gene.

**Table 3:** Stability ranking of the five candidate reference genes of *Aphelenchoides besseyi* calculated by RefFinder

Stability ranking	Four stages in population N10			Four stages in population S24		Mixed-stage nematodes of the four populations		Average ranking	
	Genes	Stability value		Genes	Stability value	Genes	Stability value	Genes	Ranking
1	*UBC*	1.414		*UBC*	1.414	*UBC*	1.189	*UBC*	1
2	*actin*	2.213		*18S rRNA*	1.565	*GAPDH*	1.414	*18S rRNA*	3.33
3	*18S rRNA*	2.711		*GAPDH*	2.280	*α-tubulin*	3.000	*actin*	3.33
4	*α-tubulin*	2.991		*actin*	4.000	*actin*	4.000	*GAPDH*	3.33
5	*GAPDH*	3.976		*α-tubulin*	5.000	*18S rRNA*	5.000	*a-tubulin*	4

## Discussion

qPCR has been commonly used for gene expression pattern analysis due to its high speed, sensitivity and specificity. However, as the technology is applied more and more widely, its shortcomings have also been gradually understood [33]. Due to its high sensitivity, the relative expression level of target genes may be significantly affected by the choice of wrong reference genes. The employment of an unstable expressed reference gene might result in wrong and invalid conclusions. Thus, it is crucial to select appropriate reference genes for stable and reproducible gene expression measurement in RT-qPCR experiments. At present, *18S rRNA* is the most commonly used reference gene for *A. besseyi*. However, the results of this study indicated that *18S rRNA* wasn’t the optimal reference gene among the five candidates.

In this study, the four candidate reference genes, *actin*, *GAPDH*, *UBC,* and *a-tubulin* which have been extensively researched in other species were cloned for the first time from *A. besseyi*. *Actin* is an important cytoskeleton protein involved in the process of cell secretion, phagocytosis, migration, cytoplasmic flow and cytoplasmic separation (Fu et al. [34]). *GAPDH* is an enzyme involved in the formation of ATP in the glycolysis reaction and is expressed in almost all tissues [35]. *UBC* is involved in the basic biochemical metabolism of organisms and is an important protein degradation enzyme [36]. *a-tubulin* is a microtubule-like protein that plays an indispensable role in the maintenance of cell shape, motility and intracellular material transport [37]. These genes are all basic components of organelles and necessary for the maintenance of life activities of the organism or participate in basic biochemical metabolic processes. In theory, they can be stably expressed in all cells and physiological conditions, suggesting that they should be ideal reference genes. In this study, we cloned *actin*, *GAPDH*, *UBC* and *α-tubulin* fragments based on the data from the transcriptome database of *A. besseyi.* The expression levels of these four genes and *18S rRNA* under three experimental conditions were determined by RT-qPCR and their stabilities were analyzed by ΔCt, geNorm, NormFinder and RefFinder. The analysis results of ΔCt indicated that *UBC* was the most suitable candidate reference gene while *α-tubulin* was the least suitable reference gene; The analysis results of geNorm indicated that *UBC* was the most suitable candidate reference gene while *18S rRNA* was the least suitable reference gene; The analysis results of NormFinder indicated that *UBC* was the most suitable candidate reference gene while *a-tubulin* was the least suitable reference gene; The analysis results of RefFinder indicated that *UBC* was the most suitable candidate reference gene while *a-tubulin* was the least suitable reference gene. The results of different softwares used to analyze the stability of the candidate genes were inconsistent because the algorithm of each softwares was different. Therefore, the use of multiple softwares for screening the reference genes can avoid the bias caused by the use of single software, and it is necessary to comprehensively consider the results of various analysis methods to select the optimal reference gene. Although the algorithms of the four methods differ, they all calculated that *UBC* was the most suitable candidate reference gene under the three experimental conditions, suggesting that *UBC* could be an ideal reference gene in RT-qPCR experiments for *A. besseyi*. The analysis results of three softwares including ΔCt, NormFinder and RefFinder deduced that *18S rRNA* was the second best candidate reference gene, whereas geNorm deduced it as the least suitable candidate reference gene.

Interestingly, the results of all four analysis softwares deduced that *18S rRNA* was the least stable reference gene under the experimental conditions of mixed-stage nematodes of different populations. The removal of the mixed-population data resulted in an average ranking increase of 1, 0.67, 1 and 0.83 in the four software of *18S rRNA*, respectively, suggesting the expression stability of *18S rRNA* between populations is not as good as it is within populations. It has been reported that ribosomal subunit copy number of *A. besseyi* have differences and intra-individual variation [18, 19], implying that variable results are easily produced in any experiment which includes mixed populations and that any ribosomal target has limited use when studying between species or even between populations of nematodes where the copy number of the ribosomal subunit per individual has not been previously established. By contrast, the copy number of the ribosomal subunit per individual within a population is likely to be consistent. This may well explain the results that the expression variation of *18S rRNA* is low in a single population and large in different populations in this study. Therefore, although *18S rRNA* is a traditional reference gene in RT-qPCR analysis for *A. besseyi*, it is not the optimal reference gene. The shortcomings of *18S rRNA*, including high expression level, variable copy number and distinctive gene types, limit it as an ideal reference gene. Using *18S rRNA* as a reference gene in RT-qPCR experiments is likely to lead to inaccurate results.

In another paper published by our group, *eIF5A* was tested as the best reference genes for *R. similis* [10]. Both papers analyzed the expression stability of *18 s rRNA*, *actin*, and *α-tubilin* in two nematode species. The difference between them was that in the previous paper the expression stability of *Rps21*, *eIF5A*, *UBI*, and *β*-PP1 in *R. similis* were analyzed while the expression stability of *GAPDH* and *UBC* in *A. besseyi* were analyzed in this paper. Some different candidate reference genes were chosen between these two papers because even the same reference gene can have very different expression level in different nematode species, and not all reference genes are suitable for different nematode species, therefore, cloning and searching for more reference genes are more beneficial to the follow-up research. In the preliminary screening work of this paper, we have also selected *eIF5A* of *A. besseyi* as a candidate gene, however, the preliminary results showed that its expression level was too low in *A. besseyi*, and its ct values were both greater than 30 in different populations and different developmental stages (data not shown), indicating that it cannot work as an ideal reference gene. Moreover, due to the low expression of *eIF5A*, its ct value cannot be detected after gradient dilution, which makes its amplification curve and amplification efficiency (E value) unavailable. Based on this, we gave up further analysis of *eIF5A* in subsequent studies. It is worth noting that *eIF5A* is moderately expressed in *R. similis*, which was different from that of *A. besseyi*, this fact confirms the above idea that even the same reference gene can have quite different expression level in different nematode species.

Although we have found a better reference gene than the one commonly used—*18S rRNA* in this study, only five candidate reference genes were analyzed under three experimental conditions, thus, whether there is a better reference gene than *UBC* remains to be further studied.

## Conclusions

In this study, four software programs, including ΔCt, geNorm, NormFinder and RefFinder, were used to analyze the expression stability of five candidate genes under three experimental conditions, the result revealed that *UBC* was an ideal reference gene in all experimental conditions, indicating *UBC* could be a suitable reference gene in RT-qPCR experiments for *A. besseyi*.

## Electronic supplementary material

Below is the link to the electronic supplementary material.

Supplementary file1 (RAR 2912 kb)

Supplementary file2 (DOCX 15 kb)

## Data Availability

The transcriptome database used in this study are available on Sequence Read Archive (SRA) (accession number: SRR4002929 and SRR4002930). The cDNA sequences of the five reference genes are available on GenBank: *actin* (KY992856), *GAPDH* (KY992853), *UBC* (KY992854), *α-tubulin* (KY992855) and *18 s rRNA* (AY508035.1). The original data of the real-time PCR experiments are available on Supplementary material files. geNorm: (https://genorm.cmgg.be/). NormFinder v20:(https://www.moma.dk/normfinder-software/). ΔCt and RefFinder: (https://www.heartcure.com.au/reffinder/?type=reference).
