# Supplementary material for: Identification and validation of reference genes for real-time RT-PCR in Aphelenchoides besseyi
Source: Mol Biol Rep. 2020 May 28;47(6):4485–94. doi: 10.1007/s11033-020-05547-8 (PMC7295731; doi:10.1007/s11033-020-05547-8)
Supplement: Supplementary file 2 — Supplementary file2 (DOCX 15 kb) [file 11033_2020_5547_MOESM2_ESM.docx]

Figure S1 PCR analysis of positive clones of the four candidate reference genes of *Aphelenchoides besseyi* 1: *actin* 2: *GAPDH* 3: *UBC* 4: *α-tubilin* M: DS2000 DNA marker

Figure S2 Prediction of conserved domains of the four candidate reference genes of *Aphelenchoides besseyi* A-D are gene fragments of *actin*, *GAPDH*, *UBC* and *α-tubulin* of *A.besseyi*

Figure S3 qPCR melting curves analysis of the five candidate reference genes. A-E are melting curves of *18S rRNA, actin*, *GAPDH*, *UBC* and *α-tubulin* of *A.besseyi*

Figure S4 qPCR products of the five candidate reference genes of *Aphelenchoides besseyi* 1:*18S rRNA* 2: *actin* 3: *GAPDH 4: UBC 5*: *α-tubilin* M: DS2000 DNA marker
